# The Acute Effects of Morning Bright Light on the Human White Adipose Tissue Transcriptome: Exploratory Post Hoc Analysis

**DOI:** 10.3390/clockssleep7030045

**Published:** 2025-08-27

**Authors:** Anhui Wang, Jeroen Vreijling, Aldo Jongejan, Valentina S. Rumanova, Ruth I. Versteeg, Andries Kalsbeek, Mireille J. Serlie, Susanne E. la Fleur, Peter H. Bisschop, Frank Baas, Dirk J. Stenvers

**Affiliations:** 1 Department of Endocrinology and Metabolism, Amsterdam Gastroenterology Endocrinology Metabolism (AGEM), Amsterdam University Medical Center (UMC), University of Amsterdam, Meibergdreef 9, 1105 AZ Amsterdam, The Netherlands; a.wang@amsterdamumc.nl (A.W.); rumanovavalentina@gmail.com (V.S.R.); m.j.serlie@amsterdamumc.nl (M.J.S.); p.h.bisschop@amsterdamumc.nl (P.H.B.); 2 Laboratory of Endocrinology, Department of Laboratory Medicine, Amsterdam University Medical Center (UMC), University of Amsterdam, Meibergdreef 9, 1105 AZ Amsterdam, The Netherlands; s.e.lafleur@amsterdamumc.nl; 3 Netherlands Institute of Neuroscience (NIN), 1105 BA Amsterdam, The Netherlands; 4 Department of Clinical Genetics, Leiden University Medical Center, 2333 ZA Leiden, The Netherlands; j.p.vreijling@lumc.nl (J.V.); frank.baas.nl@gmail.com (F.B.); 5 Laboratory of Endocrinology, Department of Clinical Chemistry, Amsterdam University Medical Center (UMC), University of Amsterdam, 1105 AZ Amsterdam, The Netherlands; a.jongejan@amsterdamumc.nl; 6 Amsterdam Public Health, Methodology, 1105 AZ Amsterdam, The Netherlands; 7 Amsterdam Institute for Immunology and Infectious Diseases, 1105 AZ Amsterdam, The Netherlands; 8 Yale School of Medicine, Section of Endocrinology, New Haven, CT 06520, USA

**Keywords:** bright light, gene set enrichment, obesity, RNA sequencing, transcriptome, white adipose tissue (WAT)

## Abstract

The circadian rhythm of the central brain clock in the suprachiasmatic nucleus (SCN) is synchronized by light. White adipose tissue (WAT) is one of the metabolic endocrine organs containing a molecular clock, and it is synchronized by the SCN. Excess WAT is a risk factor for health issues including type 2 diabetes mellitus (DM2). We hypothesized that bright-light exposure would affect the human WAT transcriptome. Therefore, we analyzed WAT biopsies from two previously performed randomized cross-over trials (trial 1: *n* = 8 lean, healthy men, and trial 2: *n* = 8 men with obesity and DM2). From 7:30 h onwards, all the participants were exposed to either bright or dim light. Five hours later, we performed a subcutaneous abdominal WAT biopsy. RNA-sequencing results showed major group differences between men with obesity and DM2 and lean, healthy men as well as a differential effect of bright-light exposure. For example, gene sets encoding proteins involved in oxidative phosphorylation or respiratory chain complexes were down-regulated under bright-light conditions in lean, healthy men but up-regulated in men with obesity and DM2. In addition to evident group differences between men with obesity and DM2 and healthy lean subjects, autonomic or neuroendocrine signals resulting from bright-light exposure also differentially affect the WAT transcriptome.

## 1. Introduction

Light exposure affects the neuroendocrine control of human energy metabolism [1]. Environmental light activates the photopigment melanopsin in the intrinsically photosensitive retinal ganglion cells (ipRGCs), which also receive input from rods and cones [2]. These ipRGCs mediate the photic entrainment of the central brain clock in the suprachiasmatic nucleus (SCN), located in the anterior hypothalamus. Subsequently, peripheral clocks in various tissues are entrained by the timing signal from the SCN via direct pathways, such as the autonomic nervous system and hormones, as well as indirect pathways, such as the feeding–fasting cycle, the sleep–wake cycle, and body temperature rhythms [3,4,5]. The molecular basis of the circadian timing system is the transcriptional–translational feedback loop (TTFL), which primarily encompasses the positive clock genes circadian locomotor output cycles kaput (*CLOCK*) and brain and muscle Arnt-like protein 1 (*BMAL1*) and the negative clock genes Period (*PER1*, *PER2*, and *PER3*) and Cryptochrome (*CRY1* and *CRY2*) [6].

White adipose tissue (WAT) is not only a means of energy storage but also an endocrine organ that contains a molecular clock [7]. Specific human fat depots such as visceral WAT or subcutaneous WAT have unique roles. Excess visceral adipose tissue in obese individuals contributes to the development of insulin resistance, diabetes, and metabolic syndrome, whereas subcutaneous WAT exerts a protective effect against metabolic abnormalities [8,9]. Even within the subcutaneous compartment, distinct depots may have different metabolic profiles. For example, the higher expression of the Homeobox (*HOX*) gene *HOXA5* and nuclear receptor subfamily 2 group F member 1 or Coup-TF1 (*NR2F1*), two molecular signatures of visceral adipocytes, in the abdominal subcutaneous WAT indicates that the abdominal subcutaneous adipose depot could be more like visceral adipose tissue than the gluteal subcutaneous fat depot [10].

Under physiological conditions, the central and peripheral circadian clocks oscillate in synchrony with the external light/dark (LD) cycle, sleep/wake behavior, and feeding/fasting behavior, contributing to optimal metabolic health [5]. However, according to the circadian disruption hypothesis, metabolic health will be compromised if this synchrony is disturbed. For instance, studies have shown that exposure to artificial light at night impacts the circadian rhythms of energy metabolism across many species, including humans [11,12,13], and the effect of light exposure at night is intensity-dependent [11]. In addition, chronic exposure to light at night has been associated with an increased prevalence of obesity and type 2 diabetes mellitus (DM2) in multiple cohort studies [14,15,16]. In addition, we previously showed that ambient light intensity acutely affects human plasma glucose and triglyceride levels in men with obesity and DM2 [17]. Moreover, obesity and low-grade inflammation may be involved in a vicious cycle with a disturbed WAT clock rhythm, leading to worsening insulin resistance in individuals with obesity and DM2 [18,19]. Therefore, in this exploratory post hoc analysis of samples collected in a previous study [17], we hypothesized that light exposure may acutely affect the human WAT transcriptome. We studied two groups of individuals: we studied healthy men to understand the transcriptomic effects of light under physiological conditions, and we studied men with obesity and DM2 to understand the effects of light under conditions of coexisting obesity, insulin resistance, and a disturbed WAT clock.

## 2. Results

### 2.1. Consistent Differences in the WAT Transcriptome Between Men with Obesity and DM2 and Lean, Healthy Men

**Figure 1:**
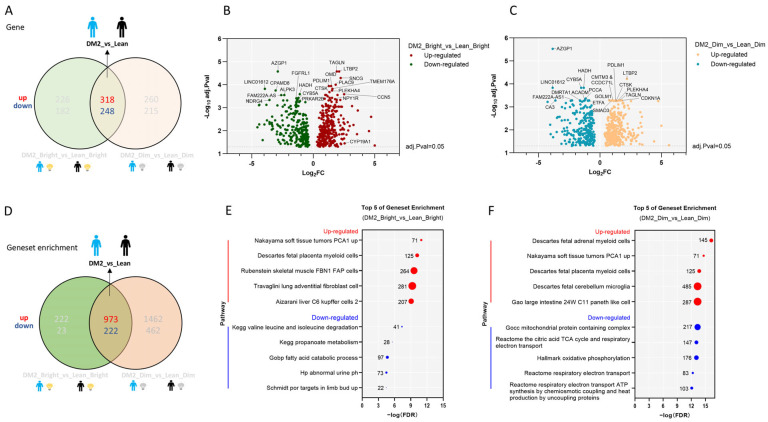
Independent of light exposure, there is a major difference in the WAT transcriptome between men with obesity and DM2 and lean, healthy men. (**A**) The number of up-regulated and down-regulated genes in men with obesity and DM2 compared to those in lean, healthy men. (**B**) A volcano plot showing the consistently up- or down-regulated genes between men with obesity and DM2 and lean men only based on the negative log10-adjusted *p* value and log2FC under bright light conditions. Names of the top 10 genes are indicated. (**C**) A volcano plot showing the consistently up- or down-regulated genes between men with obesity and DM2 and lean men only based on the negative log10-adjusted *p* value and log2FC under dim light conditions. Names of the top 10 genes are indicated. (**D**) The differences in gene set enrichment between men with obesity and DM2 compared to lean, healthy men. (**E**) The top-5-ranking consistently up- or down-regulated gene sets in men with obesity and DM2 compared to lean men only based on the FDR under bright light conditions. (**F**) The top-5-ranking consistently up- or down-regulated gene sets in men with obesity and DM2 compared to lean men only based on the FDR under dim light conditions. The numbers next to the red or blue solid circles in (**E**,**F**) indicate the number of genes enriched.

We observed significant differences in gene expression between men with obesity and DM2 and lean, healthy men (Figure 1A–C). Independent of light exposure (thus, these genes are up- or down-regulated under both dim and bright-light conditions), 318 genes were found to be up-regulated and 248 genes down-regulated in the obesity-and-DM2 group compared to the lean, healthy group, using an adjusted *p* value = 0.05 and a log2 fold change (FC) = 0 as cut-offs (Figure 1A–C, Supplementary Table S1).

The top five consistently up-regulated gene sets in men with obesity and DM2 compared to lean men were mostly related to inflammation, as can be inferred from the large number of inflammatory genes (e.g., genes related to monocyte/macrophage/lymphocyte function, complement genes, and immunoglobulin genes) that are significantly up-regulated in these gene sets. In contrast, down-regulated gene sets were mostly related to the citric acid cycle and the respiratory chain (Figure 1D–F).

### 2.2. Effects of Bright-Light Exposure on the WAT Transcriptome

#### 2.2.1. There Is No Consistent Effect of Bright Light on the WAT Transcriptome Among Men with Obesity and DM2 and Lean, Healthy Men

**Figure 2:**
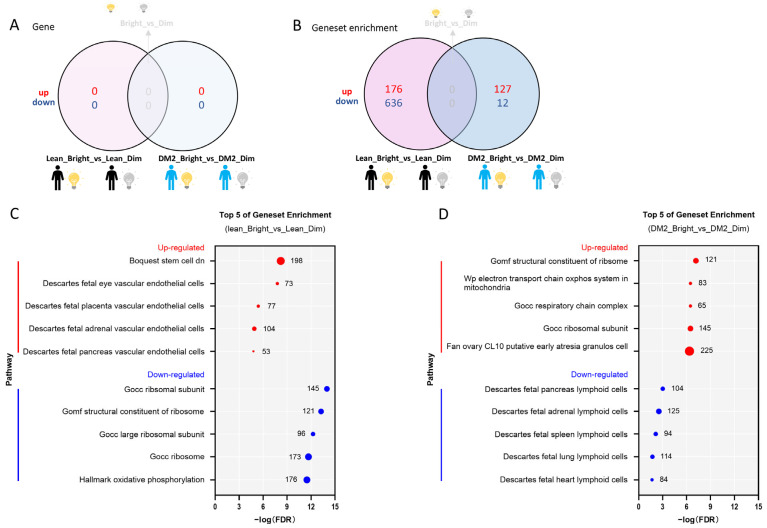
Bright light affects gene sets in men with obesity and DM2 and lean, healthy men. (**A**) The effect of bright light on gene expression in both men with obesity and DM2 and lean men compared to the effect of dim light. (**B**) The effect of bright light on gene sets in both men with obesity and DM2 and lean men in comparison to the effect of dim light. (**C**) The top 5 bright-light-affected up- and down-regulated gene sets in lean, healthy men. (**D**) The top 5 bright-light-affected up- and down-regulated gene pathways in men with obesity and DM2. The numbers next to the red and blue solid circles in (**C**,**D**) indicate the number of genes in the enrichment.

There were no single genes or gene sets differentially expressed due to bright-light exposure in comparison to dim light with a consistent pattern among the men with obesity and DM2 and the lean individuals after analysis using an adjusted *p* value = 0.05 and a log2FC = 0 as cut-offs (Figure 2A).

#### 2.2.2. The Effect of Bright Light on the WAT Transcriptome in Men with Both Obesity and DM2 and Lean, Healthy Men

Bright light did not cause differential expression of genes in either the men with obesity and DM2 or the lean men (Figure 2A). However, by combining the contributions of the various genes (i.e., the direction of change, log2FC values, and significance levels) constituting a pathway, we detected changes at the gene-set-level and discerned significant changes in pathways between the tested conditions. In fact, we found that bright light resulted in many differentially expressed gene sets among men with obesity and DM2 and among lean, healthy men (Figure 2B). The gene sets up-regulated due to bright light in the lean men included gene sets involved in vascular endothelial cell function, whereas the gene sets down-regulated due to bright light in lean men were involved in ribosome formation and oxidative phosphorylation (Figure 2C). In stark contrast, similar gene sets involved in ribosome formation, the mitochondrial oxidative phosphorylation system, and the respiratory chain complex were up-regulated due to bright light in men with obesity and DM2, while gene sets down-regulated due to bright light in men with obesity and DM2 were involved in lymphoid cell function (Figure 2D).

#### 2.2.3. Effects of Bright or Dim Light on the WAT Transcriptome Differ Between Men with Obesity and DM2 and Lean, Healthy Men

**Figure 3:**
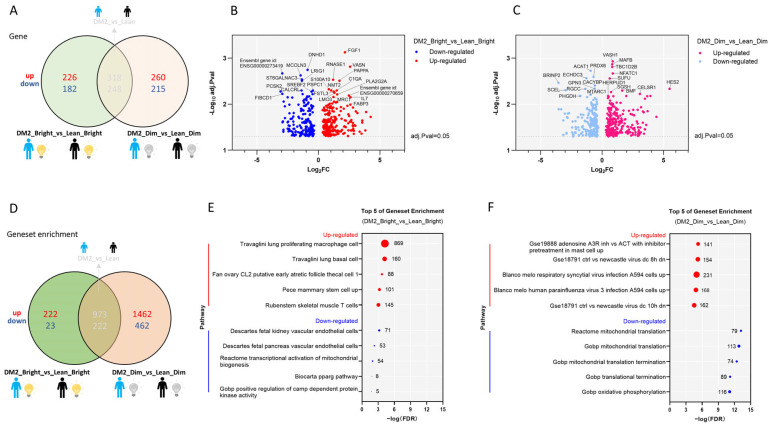
Under both bright and dim light, there are significant differences in the WAT transcriptome between men with obesity and DM2 and lean, healthy men. (**A**) The differentially expressed genes in men with obesity and DM2 in comparison to lean men under both bright and dim light. (**B**) A volcano plot showing the up- or down-regulated genes affected by bright-light exposure between men with obesity and DM2 and lean, healthy men. Names of the top 10 genes are indicated. (**C**) A volcano plot showing the up- or down-regulated genes affected by dim-light exposure between men with obesity and DM2 and lean men. Names of the top 10 genes are indicated. (**D**) The difference in gene sets in men with obesity and DM2 compared to lean men under both bright and dim light conditions. (**E**) The top 5 up- and down-regulated gene sets affected by bright-light exposure in men with obesity and DM2 in comparison to lean men. (**F**) The top 5 up- and down-regulated gene sets affected by dim-light exposure in men with obesity and DM2 in comparison to lean men. The numbers next to the red and blue solid circles in (**E**,**F**) indicate the number of genes in the enrichment.

Under bright light conditions, there were 226 genes up-regulated and 182 genes down-regulated in men with obesity and DM2 compared to lean, healthy men, as found using an adjusted *p* value = 0.05 and a log2FC = 0 as cut-offs (Figure 3A). Under dim light conditions, 260 genes were up-regulated and 215 genes were down-regulated in men with obesity and DM2 compared to lean, healthy men (Figure 3A–C). Notably, in men with obesity and DM2, the genes up-regulated due to bright light include fibroblast growth factor 1 (*FGF1*), vasorin (*VASN*), ribonuclease A family member 1 (*RNASE1*), pappalysin 1 (*PAPPA*), Interleukin 7 (*IL7*), and fatty acid binding protein 3 (*FABP3*) (Figure 3A–C, Supplementary Table S2).

Gene set enrichment analysis revealed that under bright light conditions, multiple gene sets were differentially affected by bright light in the men with obesity and DM2 compared to the lean men (Figure 3D). Under bright light conditions, the top 5 up-regulated gene sets in men with obesity and DM2 are involved in immune cell functioning, whereas the down-regulated gene sets are related to vascular endothelial cells, mitochondrial biogenesis, and kinase regulation (Figure 3E). Under dim light conditions, the up-regulated gene sets in men with obesity and DM2 are also related to immunity, whereas the down-regulated gene sets are involved in mitochondrial translation and translation termination (Figure 3F).

## 3. Discussion

In this study, we explored the effect of light exposure on the abdominal subcutaneous WAT transcriptome in lean, healthy men as well as men with obesity and DM2. We identified consistent differences, independent of light exposure, between men with obesity and DM2 and healthy, lean subjects. Early-morning exposure to bright light clearly affected the WAT transcriptome, but these effects were very different between men with obesity and DM2 and healthy, lean men.

Obesity is a chronic complex disease and can result in cardiometabolic complications via systemic inflammation, endothelial dysfunction, insulin resistance, and dyslipidemia [20,21]. We identified genes and gene sets that are consistently different between men with obesity and DM2 and lean men, independent of light exposure. The consistently up-regulated gene sets in men with obesity and DM2 in comparison to lean men are related to inflammation, which is in line with previous studies [22,23]. Our data are also in line with previously identified up-regulated genes such as the cytochrome P450 family 19 subfamily A member 1 (*CYP19A1*) gene [18], which encodes the aromatase that catalyzes the conversion of the C19 androgens androstenedione and testosterone to the aromatic C18 estrogens estrone and estradiol, respectively. Supraphysiological estradiol concentrations may contribute to insulin resistance [24,25,26,27,28]. The other up-regulated genes in men with obesity and DM2 were (1) latent transforming growth factor beta binding protein 2 (*LTBP2*), which was previously shown to also be present in elevated levels in the ligamentum flavum of patients with diabetes mellitus [29], as well as in the bone marrow mesenchymal stem cell secretome of Zucker diabetic fatty rats [30], and is a candidate diagnostic marker for diabetic peripheral neuropathy [31]; (2) transgelin (*TAGLN*), which encodes an actin-binding protein found in fibroblasts and smooth muscle, also known as smooth muscle protein 22α (SM22α) [32], and whose overexpression can induce the dysfunction of fetal endothelial colony-forming cells from gestational diabetic pregnancies [32,33]; (3) synuclein gamma (*SNCG*), which has a role in metabolic physiology and adipocyte fuel homeostasis as a peroxisome proliferator-activated receptor γ (PPARγ) target [34]; (4) cellular communication network factor 5 (*CCN5*), also named WNT1 inducible signaling pathway protein 2 (*WISP2*), which encodes for a protein that is found at high serum levels in patients with DM2 and is positively correlated with inflammatory cytokines (IL6 and TNF-α) [35]; and (5) neuropeptide Y receptor Y1 (*NPY1R*), which encodes for one of the receptors of neuropeptide NPY. NPY may impair the insulin sensitivity of adipocytes by inhibiting insulin-stimulated glucose uptake [36] and is involved in β-cell failure, contributing to the pathophysiology of DM2 [37,38].

Since environmental light information processed by the hypothalamus can reach the WAT via the autonomic nervous system as well as via endocrine signals [1,39], we hypothesized that bright-light exposure would affect the WAT transcriptome. Despite the absence of single genes that were consistently up- or down-regulated by bright-light exposure in both participant groups, we did identify some gene sets that were affected by bright-light exposure in both men with obesity and DM2 and lean, healthy men. For example, in lean, healthy men, one of the most affected gene sets down-regulated by bright light is related to mitochondrial oxidative phosphorylation. Remarkably, in men with obesity and DM2, the respiratory chain complex in mitochondria was up-regulated after bright-light exposure. The genes from this gene set of mitochondrial oxidative phosphorylation include (1) NADH:ubiquinone oxidoreductase subunit A7 (*NDUFA7*), encoding for a subunit of the NADH:ubiquinone oxidoreductase (complex I) that plays a role in the transfer of electrons from NADH to the respiratory chain, and (2) cytochrome c oxidase assembly homolog COX15 (*COX15*), encoding for a protein of COX assembly factors, the deficiency of which will lead to impaired electron transport and oxidative phosphorylation. Both these genes were up-regulated due to light in lean, healthy men but down-regulated in men with obesity and DM2. Not only the transcriptomic but also the metabolic effects of bright light were different between men with obesity and DM2 and healthy, lean men, as described in the initial study [17]. Bright light increased fasting and postprandial plasma glucose levels in men with obesity and DM2 but not in lean, healthy men. In contrast, in lean men, fasting and postprandial plasma triglyceride levels were increased due to bright light, while in men with obesity and DM2, only postprandial triglycerides were affected. It has been suggested that increased levels of triglycerides are a risk factor for the development in insulin resistance [40]. Taken together, if these acute transcriptomic effects can be extrapolated to long-term protein effects, both the down-regulated oxidative phosphorylation and the up-regulated triglycerides indicate that too much light exposure might increase the risk of insulin resistance development in the long term in lean, healthy individuals. Indeed, epidemiological studies have shown associations between light pollution and incidence of obesity and DM2 [14,15,16].

In addition to the gene sets and genes that were affected by light, with an opposite effect between the groups, we identified many genes and gene sets that were differentially affected by light between the men with obesity and DM2 and the lean, healthy men. Gene sets involved in immune function, such as gene sets the lung proliferating macrophage cell enrichment and skeletal muscle T cell enrichment, were among the gene sets most up-regulated by light in men with obesity and DM2. Considering the role of pro-inflammatory macrophages in adipose tissue in the pathophysiology of adipocyte insulin resistance and ultimately the development of DM2, these effects of light on the WAT transcriptome may provide a potential explanation of the association between light pollution and the incidence of obesity and diabetes mellitus [14,15,16], although we have to note that we exposed people to light during the early-light phase of the diurnal light–dark cycle, and the effects of light may be time-dependent [11]. The individual genes up-regulated by bright light in men with obesity and DM2 condition include (1) FGF1, which is involved in the regulation of insulin sensitivity and glucose and lipid metabolism as the downstream target of PPARγ [41]; (2) FABP3, which is the response gene of PPARγ and coordinates cellular lipid trafficking and signaling [42,43]; (3) VASN, which encodes a transforming growth factor beta-binding protein that is expressed in vascular smooth muscle cells [44] and is present in elevated quantities in plasma from people with diabetic nephropathy [45]; (4) RNASE1, which may indirectly regulate insulin production by affecting the stability of insulin mRNA [46]; (5) PAPPA, which encodes a secreted protease whose main substrate is insulin-like growth factor binding protein, and circulating PAPPA protects against the development of pre-diabetes and DM2 [47]; (6) complement C1q A chain (C1QA), which is one of the genes encoding C1q, with C1QA protein being involved in the progression of DM2 [48] and showing increased expression in visceral adipose tissue of DM2 patients [49]; (7) follistatin like 3 (FSTL3), which encodes a glycoprotein that is suggested to be involved in the development of insulin resistance and DM2 [50,51,52]; (8) phospholipase A2 group IIA (PLA2G2A), which encodes a protein located in adipose tissue immune cells and may be involved in the development of adiposity and metabolic syndrome [53]; and (9) IL7, which is an important proinflammatory mediator [54,55].

In conclusion, we have shown that exposure to bright light versus dim light differentially affects the WAT transcriptome. The effects were different between lean subjects and men with obesity and DM2. This may be related to the observed association between light pollution and the incidence of obesity and DM2 [14,15,16].

However, a major limitation of the current study is that the original trials were two separate trials that were not designed to allow a direct comparison between lean people and obese people with type 2 diabetes mellitus. For example, another difference between the groups is age, which may also contribute to the differences between groups. A second limitation is that we included only men, which limits the external validity of our findings. A third limitation of the present study is that we cannot rule out the fact that the differential effects of light on the plasma glucose and triglyceride levels may have partly mediated the differential effect on the WAT transcriptomic landscape. Finally, we assessed gene expression at the tissue level. The gene expression effects should be checked at the protein level through future studies on the metabolic effects of light. Moreover, these future studies should disentangle the cell-type-specific effects of bright light using single-cell sequencing [23], which can (1) delineate distinct transcriptional signatures in specific cell types (e.g., adipocytes versus stromal vascular fraction cells or immune cell populations) and (2) reconstruct cell-type-specific signaling cascades underlying light-induced adipose tissue adaptation. Such insights would indeed substantially advance our mechanistic understanding of how morning bright light modulates adipose tissue function at the cellular and molecular levels.

## 4. Materials and Methods

We studied abdominal subcutaneous adipose tissue samples obtained in two previously published randomized cross-over trials [17].

### 4.1. Ethical Approval

This study was performed in accordance with the principles of the Declaration of Helsinki (October 2008) and the Medical Research Involving Human Subjects Act (WMO, March 2006). The lean, healthy subject trial was registered at the Netherlands Trial Registry (NTR) as NTR3881. The obesity and DM2 subject trial was registered as NTR4645. Both trials were approved by the Institutional Review Board of the Academic Medical Center (AMC) under the approval code 2012/314, NL42188.018.12 for lean healthy participants, and 2013/259, NL46085.018.13 for participants with obesity and DM2. All participants provided written informed consent.

### 4.2. Study Participants and Screening Procedures

The details of the study, including inclusion and exclusion criteria, were published previously [17]. In brief, we performed two separate randomized cross-over trials. Study 1 included 8 lean, healthy men (subject characteristics are shown in Table 1), and study 2 included 8 men with obesity and DM2 (subject characteristics are shown in Table 2). Participants with DM2 refrained from taking metformin for 3 days before each admission. Prior to entering the research unit, participants maintained a normal sleep–wake schedule and recorded sleep–wake rhythms in a sleep–wake diary for 5 days while wearing an Actiwatch to verify adherence [17].

### 4.3. Study Design

**Figure 4:**
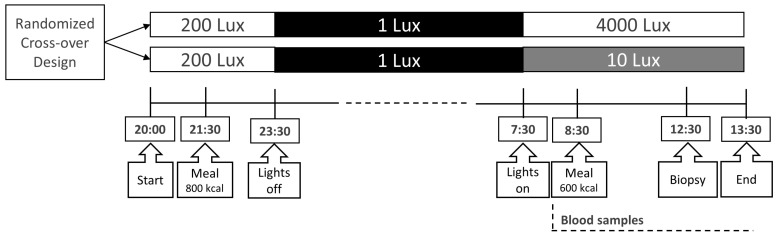
Study protocol. This randomized cross-over design was employed in both trials. Blood samples were taken frequently [17]. An adipose tissue biopsy was performed 4 h after the meal. Figure was reproduced from reference (Versteeg et al.) [17].

Participants were admitted to the clinical research unit for a 16 h protocol twice with a 1-week washout period (Figure 4) [17]. In view of the continued and unchanged regular sleep/wake and light/dark rhythms in this week, we did not expect the effects of 6 h bright- or dim-light exposure to be sustained longer than a week [56,57]. Participants fasted for 1 h before admission, and they received a standard 800 kilocalorie (kcal) meal at 21:30 h. Thereafter, they remained under normal room light (200 lux) until lights-out at 23:30 h and slept in the dark (1 lux). At 07:30 h, lights were turned on again at either a bright (4000 lux) or dim (10 lux) level. Initially, participants were randomly assigned to either bright- or dim-light exposure via a randomization table. An intravenous cannula was inserted into a peripheral arm vein at 08:00 h for frequent blood sampling. One hour after lights-on at 08:30 h, participants consumed a standard 600 kcal liquid mixed meal (400 mL Ensure Plus, Abbott Industries, Chicago, IL, USA). An adipose tissue biopsy from the subcutaneous abdominal compartment was performed 4 h after the meal.

### 4.4. Lighting Conditions

Light was emitted by EnergyLights HF3319 (Philips Consumer Lifestyle B.V., Eindhoven, The Netherlands). In the bright-light condition, two EnergyLights HF3319 (Philips, Amsterdam, The Netherlands) were placed 60 cm in front of the participant, yielding 4000 lux, which is similar to outside light levels on a cloudy day. In the control condition, participants were exposed to dim light at a level of 10 lux emitted by one EnergyLight HF3319 (Philips, Amsterdam, The Netherlands) placed in the corner of the room; this level is comparable to light levels at twilight.

### 4.5. Adipose Tissue Biopsy

At the end of each exposure period, a subcutaneous fat biopsy of the periumbilical region was performed. After local anesthesia with 5cc of 2% lidocaine, biopsies were obtained using a 15-gauge needle under continuous vacuum suction conditions. Samples were directly rinsed with 0.9% sodium chloride and subsequently added to 1 mL of Tripure Isolation Reagent (Roche, Basel, Switzerland) shaken using a TissueLyser (Qiagen, Hilden, Germany) and stored at −80 °C within 15 min after tissue sampling. After each biopsy, a continuous pressure bandage was applied to the biopsy site for 15 min to achieve hemostasis and minimize hematomas.

### 4.6. RNA Sequencing

Fat biopsies were lysed in Tripure Isolation Reagent (Roche, Basel, Switzerland). After extraction with phenol/chloroform, RNA isolation was performed using QIAsymphony RNA Kit (Ref. #931636; QIAGEN), followed by extra purification using Macherey-Nagel NucleoSpin RNA XS Kit (Ref. #740902.50) including a DNAse I treatment. RNA concentrations were measured using Nanodrop. Subsequent quality control and sequencing were performed by Genomescan (Leiden, The Netherlands). RNA Samples were processed by using the NEBNext Ultra II Directional RNA Library Prep Kit for Illumina (NEB #E7760S/L). At first, mRNA was isolated from total RNA using oligo-dt magnetic beads. After fragmentation of the mRNA, cDNA synthesis was performed. This was used for ligation with the sequencing adaptors and PCR amplification of the resulting product. Quality and yield after sample preparation were measured using Fragment Analyzer. The sizes of the resulting products were consistent with the expected size distribution (300–500 bp). Clustering and DNA sequencing were performed using NovaSeq6000 v1.5, PE150 (Illumina, San Diego, CA, USA). A concentration of 1.1 nM of DNA was used. NovaSeq control software NCS v1.7.5 was used. Image analysis, base calling, and quality checks were performed with the Illumina data analysis pipeline RTA3.4.4 and Bcl2fastq v2.20. Due to technical issues, 5 samples were lost during RNA sequencing, and 1 sample was excluded during data processing (see statistical analysis below). This resulted in 5 paired samples (from the same individual) and 2 unpaired samples from the lean, healthy men and 6 paired samples and 2 unpaired samples from the obese men with DM2.

### 4.7. Statistical Analysis

Reads were subjected to quality control (FastQC, dupRadar [58], Picard Tools), trimmed using Trimmomatic v0.39 [59], and aligned to the GRCh38 (v105) genome using HISAT2 (v2.2.1) [60]. Counts were obtained via HTSeq (v1.99.2) [61] using the corresponding GTFs. During quality control, one extreme outlier was observed in the MDS plot, and this sample was subsequently excluded from further analysis. Statistical analyses were performed using the edgeR (v3.34.1) [62] and limma (v3.48.3)/voom [63] R packages. All genes with no counts in any of the samples were removed, whilst genes with more than 2 reads in at least 6 of the samples were kept. Count data were transformed into log2-counts per million (logCPM), normalized by applying the trimmed mean from the M-values method [62] and precision-weighted using voom [64]. Differential expression was assessed using an empirical Bayes-moderated t-test within limma’s linear model framework including the precision weights estimated by voom. Pairing of samples was accounted for using the duplicate Correlation function of limma. Resulting P values were corrected for multiple testing using the Benjamini–Hochberg false-discovery rate (FDR). Genes were re-annotated via biomaRt using the Ensembl genome database (v106). Gene set enrichment was performed with MSigDB gene sets (v7.4) using the CAMERA approach as implemented in limma. The resulting DEGs, Venn diagrams, expression plots, and gene set enrichment results were shown in an in-house-made Shiny app. The analysis was performed using R v4.1.0 and Bioconductor v3.13.

### 4.8. Power Calculation

In the original study, power calculation was based on the primary outcome, i.e., fasting, and postprandial glucose levels [17]. In addition, based on our previous studies and those conducted by others [18,65,66], we knew that a sample size of eight subjects would be sufficient to detect biologically relevant differences in adipose tissue gene expression using RNA sequencing through a repeated-measures design.

## Figures and Tables

**Table 1:** Characteristics of lean, healthy men [17].

Characteristic	
Age, years	23 (21–24)
BMI, kg/m^2^	22 (22–23)
HbA_1c_, %	5.2 (5.1–5.5)
HbA_1c_, mmol/mol	34 (33–37)
Fasting glucose, mmol/L	5.0 (4.8–5.0)
Fasting insulin, pmol/L	40 (12–62)
Sleep parameters from diary	
Onset of sleep, time	23:48 h ± 13 min
End of sleep, time	08:02 h ± 6 min

Normally distributed data are expressed as means ± standard error of the mean (SEM); non-normally distributed data are expressed as medians (25–75th percentiles).

**Table 2:** Characteristics of men with obesity and DM2 [17].

Characteristic	
Age, years	60 (54–63)
BMI, kg/m^2^	30 (28–35)
HbA_1c_, %	6.8 (6.7–8.0)
HbA_1c_, mmol/mol	51 (50–65)
Fasting glucose, mmol/L	8.4 (6.2–10)
Fasting insulin, pmol/L	119 (111–184)
Medication, *n* (%)	
Metformin ^a^	8 (100)
Lipid-lowering drugs	4 (50)
Antihypertensives	3 (37.5)
Proton pump inhibitor	1 (12.5)
Sleep parameters from diary	
Onset of sleep, time	00:05 h ± 7 min
End of sleep, time	07:40 h ± 10 min

Normally distributed data are expressed as means ± standard error of the mean (SEM); non-normally distributed data are expressed as medians (25–75th percentiles). ^a^ Metformin administration was discontinued 3 days prior to each admission.

## Data Availability

The original data presented in the study are openly available in European Genome-Phenome Archive (EGA) with accession number EGAD50000001715.

## Supplementary Materials

The following supporting information can be downloaded at https://www.mdpi.com/article/10.3390/clockssleep7030045/s1. Table S1: Consistent differences in the WAT transcriptome between men with obesity and DM2 and lean, healthy men. The top 20 consistently up- and down-regulated genes in men with obesity and DM2 compared to lean, healthy men based on the adjusted *p* value only under either bright-light-exposure or dim-light-exposure conditions. Table S2: Different effects of light on the WAT transcriptome between men with obesity and DM2 and lean, healthy men. The top 20 up- and down-regulated genes affected by either bright-light or dim-light exposure in men with obesity and DM2 compared to lean, healthy men.
