# Supplementary material for: The Acute Effects of Morning Bright Light on the Human White Adipose Tissue Transcriptome: Exploratory Post Hoc Analysis
Source: Clocks Sleep. 2025 Aug 27;7(3):45. doi: 10.3390/clockssleep7030045 (PMC12452623; doi:10.3390/clockssleep7030045)
Supplement: Supplementary file 1 [file clockssleep-07-00045-s001.zip › clockssleep-3624262-supplementary.pdf]

Supplementary Material
Supplementary tables

Table S1. Consistent differences in the WAT transcriptome between men with obesity and DM2 and lean healthy men. The top 20 consistent up- and down-regulated genes in men with obesity and DM2 compared to lean healthy men based on the adjusted P value only from either bright light exposure or dim light exposure condition.

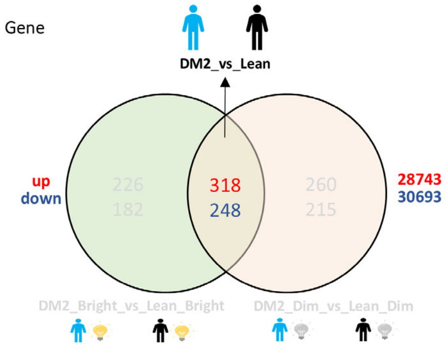

	DM2_Bright_vs_Lean_Bright				DM2_Dim_vs_Lean_Dim			
	HGNC Symbol (Gene Symbol)	log2 Fold Change	Adjusted P value (FDR)	P value	HGNC Symbol (Gene Symbol)	log2 Fold Change	Adjusted P value (FDR)	P value
Up-regulated	LTBP2	2.10	2.64e-05	2.12e-09	LTBP2	2.19	6.06e-05	6.05e-09
	TAGLN	1.92	2.64e-05	3.95e-09	PDLIM1	1.28	5.24e-04	1.83e-07
	SNCG	2.23	5.10e-05	1.02e-08	TAGLN	1.64	5.25e-04	3.30e-07
	PLAC9	1.82	1.00e-04	2.50e-08	PLEKHA4	1.54	5.25e-04	4.49e-07
	PDLIM1	1.27	1.14e-04	3.53e-08	CMTM3	1.05	5.25e-04	3.30e-07
	OMD	1.45	1.14e-04	3.97e-08	CDKN1A	1.78	5.25e-04	5.24e-07
	TMEM176A	1.56	1.51e-04	6.79e-08	CCDC71L	1.05	5.25e-04	3.07e-07
	PLEKHA4	1.53	1.77e-04	8.85e-08	GOLM1	0.77	5.25e-04	4.89e-07
	CTSK	1.32	2.08e-04	1.25e-07	SMAD3	0.93	5.25e-04	3.94e-07
	CCN5	2.51	2.57e-04	1.80e-07	CTSK	1.32	5.46e-04	6.07e-07
	CMTM3	0.98	2.77e-04	2.21e-07	C1S	1.41	5.52e-04	6.96e-07
	VSIG4	1.97	2.77e-04	2.07e-07	SLC43A3	1.13	5.52e-04	7.24e-07
	LUM	1.06	2.80e-04	2.66e-07	CCN5	2.52	5.57e-04	7.78e-07
	TSHZ2	1.17	3.18e-04	3.18e-07	NALCN	4.73	5.59e-04	8.09e-07
	TMEM176B	1.53	3.71e-04	4.09e-07	ISLR	1.89	6.56e-04	1.05e-06
	C1S	1.33	3.71e-04	3.95e-07	SNCG	1.85	7.05e-04	1.20e-06
	IFITM3	0.93	4.05e-04	5.06e-07	TMEM176A	1.49	7.82e-04	1.44e-06
	NPY1R	1.78	4.23e-04	5.48e-07	NPR3	2.27	8.74e-04	1.72e-06
	MGP	1.39	4.59e-04	6.88e-07	THBS2	1.99	8.91e-04	1.82e-06
	NPR3	2.20	4.59e-04	6.73e-07	HSPA7	1.96	9.38e-04	2.01e-06
Down-regulated	AZGP1	-2.88	2.64e-05	3.35e-09	AZGP1	-3.85	2.98e-06	1.48e-10
	LINC01612	-3.93	1.51e-04	6.05e-08	LINC01612	-3.85	1.47e-04	3.66e-08
	CPAMD8	-3.06	1.77e-04	9.74e-08	HADH	-1.32	1.47e-04	2.66e-08
	HADH	-1.09	2.57e-04	1.69e-07	CYB5A	-1.53	1.47e-04	3.52e-08
	ALPK3	-2.35	2.80e-04	2.40e-07	PCCA	-1.16	4.33e-04	1.30e-07
	FAM222A-AS1	-2.60	2.80e-04	2.64e-07	FAM222A-AS1	-2.27	5.25e-04	5.04e-07
	CYB5A	-1.20	3.71e-04	4.26e-07	ACADM	-1.24	5.25e-04	4.36e-07
	FGFRL1	-1.37	3.88e-04	4.65e-07	CA3	-3.62	5.25e-04	4.05e-07
	PRKAR2B	-1.27	4.59e-04	7.33e-07	ETFA	-0.88	5.25e-04	4.62e-07
	NDRG4	-3.56	4.59e-04	6.51e-07	DMRTA1	-1.62	5.25e-04	4.90e-07
	ACSL1	-1.27	4.59e-04	7.18e-07	RIDA	-0.99	5.25e-04	4.87e-07
	SLC1A3	-1.32	4.80e-04	8.63e-07	ALPK3	-2.38	5.46e-04	6.32e-07
	STOX1	-2.44	4.80e-04	8.27e-07	ALDH6A1	-1.31	5.46e-04	6.54e-07
	ADAMTS9-AS1	-2.12	4.80e-04	8.59e-07	HSPD1	-0.97	5.46e-04	6.42e-07
	ACADM	-1.08	5.12e-04	9.45e-07	TSPAN13	-1.61	5.52e-04	7.44e-07
	MPDZ	-1.34	5.67e-04	1.14e-06	SLC1A3	-1.45	5.94e-04	8.90e-07
	ALDH6A1	-1.15	5.67e-04	1.12e-06	COL6A6	-4.26	6.04e-04	9.35e-07
	LPIN1	-1.39	5.67e-04	1.16e-06	CPAMD8	-2.77	6.80e-04	1.12e-06
	PPP2R5A	-0.65	5.71e-04	1.23e-06	DMRT2	-2.23	7.79e-04	1.36e-06
	TSPAN13	-1.39	6.98e-04	1.57e-06	SLC16A7	-1.59	7.82e-04	1.41e-06

	DM2_Bright_vs_Lean_Bright				DM2_Dim_vs_Lean_Dim			
	HGNC Symbol (Gene Symbol)	log2 Fold Change	Adjusted P value (FDR)	P value	HGNC Symbol (Gene Symbol)	log2 Fold Change	Adjusted P value (FDR)	P value
**Up-regulated**	LTBP2	2.10	2.64e-05	2.12e-09	LTBP2	2.19	6.06e-05	6.05e-09
	TAGLN	1.92	2.64e-05	3.95e-09	PDLIM1	1.28	5.24e-04	1.83e-07
	SNCG	2.23	5.10e-05	1.02e-08	TAGLN	1.64	5.25e-04	3.30e-07
	PLAC9	1.82	1.00e-04	2.50e-08	PLEKHA4	1.54	5.25e-04	4.49e-07
	PDLIM1	1.27	1.14e-04	3.53e-08	CMTM3	1.05	5.25e-04	3.30e-07
	OMD	1.45	1.14e-04	3.97e-08	CDKN1A	1.78	5.25e-04	5.24e-07
	TMEM176A	1.56	1.51e-04	6.79e-08	CCDC71L	1.05	5.25e-04	3.07e-07
	PLEKHA4	1.53	1.77e-04	8.85e-08	GOLM1	0.77	5.25e-04	4.89e-07
	CTSK	1.32	2.08e-04	1.25e-07	SMAD3	0.93	5.25e-04	3.94e-07
	CCN5	2.51	2.57e-04	1.80e-07	CTSK	1.32	5.46e-04	6.07e-07
	CMTM3	0.98	2.77e-04	2.21e-07	C1S	1.41	5.52e-04	6.96e-07
	VSIG4	1.97	2.77e-04	2.07e-07	SLC43A3	1.13	5.52e-04	7.24e-07
	LUM	1.06	2.80e-04	2.66e-07	CCN5	2.52	5.57e-04	7.78e-07
	TSHZ2	1.17	3.18e-04	3.18e-07	NALCN	4.73	5.59e-04	8.09e-07
	TMEM176B	1.53	3.71e-04	4.09e-07	ISLR	1.89	6.56e-04	1.05e-06
	C1S	1.33	3.71e-04	3.95e-07	SNCG	1.85	7.05e-04	1.20e-06
	IFITM3	0.93	4.05e-04	5.06e-07	TMEM176A	1.49	7.82e-04	1.44e-06
	NPY1R	1.78	4.23e-04	5.48e-07	NPR3	2.27	8.74e-04	1.72e-06
	MGP	1.39	4.59e-04	6.88e-07	THBS2	1.99	8.91e-04	1.82e-06
	NPR3	2.20	4.59e-04	6.73e-07	HSPA7	1.96	9.38e-04	2.01e-06
**Down-regulated**	AZGP1	-2.88	2.64e-05	3.35e-09	AZGP1	-3.85	2.98e-06	1.48e-10
	LINC01612	-3.93	1.51e-04	6.05e-08	LINC01612	-3.85	1.47e-04	3.66e-08
	CPAMD8	-3.06	1.77e-04	9.74e-08	HADH	-1.32	1.47e-04	2.66e-08
	HADH	-1.09	2.57e-04	1.69e-07	CYB5A	-1.53	1.47e-04	3.52e-08
	ALPK3	-2.35	2.80e-04	2.40e-07	PCCA	-1.16	4.33e-04	1.30e-07
	FAM222A-AS1	-2.60	2.80e-04	2.64e-07	FAM222A-AS1	-2.27	5.25e-04	5.04e-07
	CYB5A	-1.20	3.71e-04	4.26e-07	ACADM	-1.24	5.25e-04	4.36e-07
	FGFRL1	-1.37	3.88e-04	4.65e-07	CA3	-3.62	5.25e-04	4.05e-07
	PRKAR2B	-1.27	4.59e-04	7.33e-07	ETFA	-0.88	5.25e-04	4.62e-07
	NDRG4	-3.56	4.59e-04	6.51e-07	DMRTA1	-1.62	5.25e-04	4.90e-07
	ACSL1	-1.27	4.59e-04	7.18e-07	RIDA	-0.99	5.25e-04	4.87e-07
	SLC1A3	-1.32	4.80e-04	8.63e-07	ALPK3	-2.38	5.46e-04	6.32e-07
	STOX1	-2.44	4.80e-04	8.27e-07	ALDH6A1	-1.31	5.46e-04	6.54e-07
	ADAMTS9-AS1	-2.12	4.80e-04	8.59e-07	HSPD1	-0.97	5.46e-04	6.42e-07
	ACADM	-1.08	5.12e-04	9.45e-07	TSPAN13	-1.61	5.52e-04	7.44e-07
	MPDZ	-1.34	5.67e-04	1.14e-06	SLC1A3	-1.45	5.94e-04	8.90e-07
	ALDH6A1	-1.15	5.67e-04	1.12e-06	COL6A6	-4.26	6.04e-04	9.35e-07
	LPIN1	-1.39	5.67e-04	1.16e-06	CPAMD8	-2.77	6.80e-04	1.12e-06
	PPP2R5A	-0.65	5.71e-04	1.23e-06	DMRT2	-2.23	7.79e-04	1.36e-06
	TSPAN13	-1.39	6.98e-04	1.57e-06	SLC16A7	-1.59	7.82e-04	1.41e-06

Table S2. Different effects of light on the WAT transcriptome between men with obesity and DM2 and lean healthy men. The top 20 up- and down-regulated genes affected by either bright light or dim light exposure in men with obesity and DM2 compared to lean healthy men.

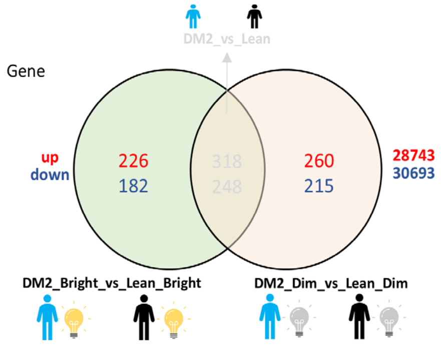

	DM2_Bright_vs_Lean_Bright				DM2_Dim_vs_Lean_Dim			
	HGNC Symbol (Gene Symbol)	log2 Fold Change	Adjusted P value (FDR)	P value	HGNC Symbol (Gene Symbol)	log2 Fold Change	Adjusted P value (FDR)	P value
Up-regulated	FGF1	2.15	0.000744	1.75e-06	VASH1	0.87	0.00117	3.44e-06
	VASN	2.57	0.001530	7.39e-06	MAFB	0.82	0.00135	4.33e-06
	RNASE1	1.19	0.002930	2.18e-05	TBC1D2B	0.82	0.00154	5.21e-06
	PAPPA	1.71	0.003100	2.42e-05	NFATC1	0.87	0.00214	1.04e-05
	S100A10	0.84	0.004720	4.62e-05	SUFU	0.62	0.00275	1.63e-05
	C1QA	1.48	0.005030	5.03e-05	HES2	5.48	0.00461	3.93e-05
	NMT2	1.03	0.005030	5.07e-05	BMF	1.63	0.00503	4.57e-05
	FSTL3	1.24	0.005290	5.62e-05	SGSH	0.73	0.00543	5.15e-05
	LMO3	1.30	0.005630	6.15e-05	CELSR1	3.07	0.00597	5.93e-05
	PLA2G2A	1.52	0.006110	7.28e-05	HERPUD1	0.49	0.00659	7.16e-05
	Ensembl gene id: ENSG00000270659	2.47	0.006480	8.02e-05	LINC00900	1.96	0.00659	7.18e-05
	MRC1	1.22	0.006960	9.23e-05	PURPL	3.92	0.00659	7.24e-05
	IL7	2.60	0.007060	9.43e-05	MITF	0.91	0.00667	7.51e-05
	FTH1P7	1.26	0.008810	1.33e-04	FAM225A	3.67	0.00667	7.47e-05
	C1QC	1.52	0.008920	1.37e-04	MEF2D	0.54	0.00687	7.95e-05
	CLIP3	1.42	0.009190	1.46e-04	SPPL3	0.48	0.00734	8.72e-05
	CRABP2	1.12	0.009630	1.63e-04	HPD	3.48	0.00754	9.07e-05
	NEXN	0.92	0.009720	1.67e-04	MAFK	0.64	0.00829	1.06e-04
	~~FABP3~~	~~2.59~~	~~0.010200~~	~~1.81e-04~~	~~ACTN1~~	~~0.65~~	~~0.00830~~	~~1.07e-04~~
	S1PR2	0.80	0.010300	1.84e-04	CD44	1.18	0.00915	1.25e-04
Down-regulated	DNHD1	-0.88	0.00179	9.73e-06	ACAT1	-0.93	0.00187	7.77e-06
	Ensembl gene id: ENSG00000273419	-2.94	0.00214	1.34e-05	PRDX6	-0.70	0.00255	1.40e-05
	MCOLN3	-1.45	0.00237	1.61e-05	ECHDC3	-1.27	0.00270	1.53e-05
	ST6GALNAC3	-1.34	0.00289	2.14e-05	BRINP2	-3.54	0.00343	2.49e-05
	LRIG1	-1.34	0.00310	2.44e-05	CACYBP	-0.58	0.00392	3.11e-05
	CALCRL	-1.37	0.00503	5.01e-05	GPN3	-0.59	0.00455	3.83e-05
	PSPC1	-0.45	0.00505	5.18e-05	RGCC	-1.35	0.00461	3.96e-05
	PCSK2	-3,08	0.00529	5.64e-05	SCEL	-2.96	0.00493	4.31e-05
	SREBF2	-0.56	0.00532	5.74e-05	MTARC1	-0.94	0.00617	6.22e-05
	FIBCD1	-2.93	0.00594	6.69e-05	PHGDH	-1.79	0.00653	6.77e-05
	BTNL9	-1.29	0.00604	7.14e-05	CIDEA	-1.76	0.00659	7.13e-05
	CHP1	-0.48	0.00604	7.11e-05	PPP1R1B	-1.22	0.00695	8.08e-05
	AGTR1	-0.75	0.00674	8.69e-05	MRPS7	-0.59	0.00734	8.76e-05
	Ensembl gene id: ENSG00000249592	-0.92	0.00677	8.86e-05	COPS5	-0.45	0.00783	9.63e-05
	MYO10	-0.89	0.00688	9.06e-05	MRPS33	-0.71	0.00803	1.01e-04
	C9	-2.50	0.00706	9.44e-05	CCT4	-0.43	0.00889	1.19e-04
	CRHBP	-2.13	0.00716	9.76e-05	UQCRH	-0.89	0.00916	1.25e-04
	ENPEP	-0.99	0.00740	1.03e-04	GPATCH11	-0.82	0.00981	1.41e-04
	LTBP1	-0.98	0.00742	1.04e-04	C1orf43	-0.41	0.00983	1.42e-04
	RBM14	-0.62	0.00764	1.08e-04	RRP9	-0.85	0.01010	1.47e-04

	DM2_Bright_vs_Lean_Bright				DM2_Dim_vs_Lean_Dim			
	HGNC Symbol (Gene Symbol)	log2 Fold Change	Adjusted P value (FDR)	P value	HGNC Symbol (Gene Symbol)	log2 Fold Change	Adjusted P value (FDR)	P value
**Up-regulated**	FGF1	2.15	0.000744	1.75e-06	VASH1	0.87	0.00117	3.44e-06
	VASN	2.57	0.001530	7.39e-06	MAFB	0.82	0.00135	4.33e-06
	RNASE1	1.19	0.002930	2.18e-05	TBC1D2B	0.82	0.00154	5.21e-06
	PAPPA	1.71	0.003100	2.42e-05	NFATC1	0.87	0.00214	1.04e-05
	S100A10	0.84	0.004720	4.62e-05	SUFU	0.62	0.00275	1.63e-05
	C1QA	1.48	0.005030	5.03e-05	HES2	5.48	0.00461	3.93e-05
	NMT2	1.03	0.005030	5.07e-05	BMF	1.63	0.00503	4.57e-05
	FSTL3	1.24	0.005290	5.62e-05	SGSH	0.73	0.00543	5.15e-05
	LMO3	1.30	0.005630	6.15e-05	CELSR1	3.07	0.00597	5.93e-05
	PLA2G2A	1.52	0.006110	7.28e-05	HERPUD1	0.49	0.00659	7.16e-05
	Ensembl gene id: ENSG00000270659	2.47	0.006480	8.02e-05	LINC00900	1.96	0.00659	7.18e-05
	MRC1	1.22	0.006960	9.23e-05	PURPL	3.92	0.00659	7.24e-05
	IL7	2.60	0.007060	9.43e-05	MITF	0.91	0.00667	7.51e-05
	FTH1P7	1.26	0.008810	1.33e-04	FAM225A	3.67	0.00667	7.47e-05
	C1QC	1.52	0.008920	1.37e-04	MEF2D	0.54	0.00687	7.95e-05
	CLIP3	1.42	0.009190	1.46e-04	SPPL3	0.48	0.00734	8.72e-05
	CRABP2	1.12	0.009630	1.63e-04	HPD	3.48	0.00754	9.07e-05
	NEXN	0.92	0.009720	1.67e-04	MAFK	0.64	0.00829	1.06e-04
	FABP3	2.59	0.010200	1.81e-04	ACTN1	0.65	0.00830	1.07e-04
	S1PR2	0.80	0.010300	1.84e-04	CD44	1.18	0.00915	1.25e-04
**Down-regulated**	DNHD1	-0.88	0.00179	9.73e-06	ACAT1	-0.93	0.00187	7.77e-06
	Ensembl gene id: ENSG00000273419	-2.94	0.00214	1.34e-05	PRDX6	-0.70	0.00255	1.40e-05
	MCOLN3	-1.45	0.00237	1.61e-05	ECHDC3	-1.27	0.00270	1.53e-05
	ST6GALNAC3	-1.34	0.00289	2.14e-05	BRINP2	-3.54	0.00343	2.49e-05
	LRIG1	-1.34	0.00310	2.44e-05	CACYBP	-0.58	0.00392	3.11e-05
	CALCRL	-1.37	0.00503	5.01e-05	GPN3	-0.59	0.00455	3.83e-05
	PSPC1	-0.45	0.00505	5.18e-05	RGCC	-1.35	0.00461	3.96e-05
	PCSK2	-3,08	0.00529	5.64e-05	SCEL	-2.96	0.00493	4.31e-05
	SREBF2	-0.56	0.00532	5.74e-05	MTARC1	-0.94	0.00617	6.22e-05
	FIBCD1	-2.93	0.00594	6.69e-05	PHGDH	-1.79	0.00653	6.77e-05
	BTNL9	-1.29	0.00604	7.14e-05	CIDEA	-1.76	0.00659	7.13e-05
	CHP1	-0.48	0.00604	7.11e-05	PPP1R1B	-1.22	0.00695	8.08e-05
	AGTR1	-0.75	0.00674	8.69e-05	MRPS7	-0.59	0.00734	8.76e-05
	Ensembl gene id: ENSG00000249592	-0.92	0.00677	8.86e-05	COPS5	-0.45	0.00783	9.63e-05
	MYO10	-0.89	0.00688	9.06e-05	MRPS33	-0.71	0.00803	1.01e-04
	C9	-2.50	0.00706	9.44e-05	CCT4	-0.43	0.00889	1.19e-04
	CRHBP	-2.13	0.00716	9.76e-05	UQCRH	-0.89	0.00916	1.25e-04
	ENPEP	-0.99	0.00740	1.03e-04	GPATCH11	-0.82	0.00981	1.41e-04
	LTBP1	-0.98	0.00742	1.04e-04	C1orf43	-0.41	0.00983	1.42e-04
	RBM14	-0.62	0.00764	1.08e-04	RRP9	-0.85	0.01010	1.47e-04
